# *RB* mutation and RAS overexpression induce resistance to NK cell-mediated cytotoxicity in glioma cells

**DOI:** 10.1186/s12935-015-0209-x

**Published:** 2015-06-05

**Authors:** Mario Orozco-Morales, Francisco Javier Sánchez-García, Irene Golán-Cancela, Norma Hernández-Pedro, Jose A. Costoya, Verónica Pérez de la Cruz, Sergio Moreno-Jiménez, Julio Sotelo, Benjamín Pineda

**Affiliations:** Laboratorio de inmunorregulación, Escuela Nacional de Ciencias Biologicas, Instituto Politecnico Nacional, Mexico, DF Mexico; Molecular Oncology Laboratory MOL, CIMUS; IDIS Departamento de Fisioloxia, Universidade de Santiago de Compostela, Av de Barcelona s/n 15782, Santiago de Compostela, Spain; Neuroimmunology and Neuro-Oncology Unit, Instituto Nacional de Neurología y Neurocirugía, Insurgentes sur 3877, 14269 Mexico City, Mexico; Neurochemistry Unit, Instituto Nacional de Neurología y Neurocirugía, Mexico, DF Mexico; Neuroradiosurgery, Instituto Nacional de Neurología y Neurocirugía, Mexico, DF Mexico

**Keywords:** Glioblastoma, Tumorigenesis, Rb, Ras, Immune evasion, Natural Killer cells

## Abstract

Several theories aim to explain the malignant transformation of cells, including the mutation of tumor suppressors and proto-oncogenes. Deletion of *Rb* (a tumor suppressor), overexpression of mutated Ras (a proto-oncogene), or both, are sufficient for *in vitro* gliomagenesis, and these genetic traits are associated with their proliferative capacity. An emerging hallmark of cancer is the ability of tumor cells to evade the immune system. Whether specific mutations are related with this, remains to be analyzed. To address this issue, three transformed glioma cell lines were obtained (Rb^−/−^, Ras^V12^, and Rb^−/−^/Ras^V12^) by *in vitro* retroviral transformation of astrocytes, as previously reported. In addition, Ras^V12^ and Rb^−/−^/Ras^V12^ transformed cells were injected into SCID mice and after tumor growth two stable glioma cell lines were derived. All these cells were characterized in terms of Rb and Ras gene expression, morphology, proliferative capacity, expression of MHC I, Rae1δ, and Rae1αβγδε, mult1, H60a, H60b, H60c, as ligands for NK cell receptors, and their susceptibility to NK cell-mediated cytotoxicity. Our results show that transformation of astrocytes (Rb loss, Ras overexpression, or both) induced phenotypical and functional changes associated with resistance to NK cell-mediated cytotoxicity. Moreover, the transfer of cell lines of transformed astrocytes into SCID mice increased resistance to NK cell-mediated cytotoxicity, thus suggesting that specific changes in a tumor suppressor (*Rb*) and a proto-oncogene (Ras) are enough to confer resistance to NK cell-mediated cytotoxicity in glioma cells and therefore provide some insight into the ability of tumor cells to evade immune responses.

## Non-English language abstract

Varias teorías pretenden explicar la transformación maligna de las células, como es la mutación de genes supresores de tumor y proto-oncogenes. La deleción de Rb (un supresor de tumor), la sobreexpresión de Ras mutado (un proto-oncogén), o ambos, son suficientes para desarrollar gliomagénesis *in vitro*, y estas características genéticas se asocian con su alta tasa de proliferación. Un rasgo distintivo del cáncer es la capacidad de las células tumorales para evadir el sistema inmune. Por lo que en este estudio analizamos si las mutaciones específicas están relacionadas con la evasión de la respuesta inmune. Para abordar esta cuestión, tres líneas celulares de glioma transformadas se obtuvieron (Rb^−/−^, Ras^V12^, y Rb^−/−^/Ras^V12^) mediante transformación retroviral de astrocitos *in vitro*, reportado anteriormente. Además, las células transformadas Ras^V12^ y Rb^−/−^/Ras^V12^ fueron inyectadas en ratones SCID y después del crecimiento del tumor se obtuvieron dos líneas celulares de glioma estables. En todas las células se determinaron la expresión génica Rb y Ras, morfología, capacidad de proliferación, expresión de MHC I, Rae1δ, and Rae1αβγδε, mult1, H60a, H60b, H60c, como ligandos para receptores de células NK, y su susceptibilidad a la citotoxicidad mediada por células NK. Nuestros resultados muestran que la transformación de los astrocitos (pérdida de Rb, la sobreexpresión de Ras, o ambos) indujo cambios fenotípicos y funcionales asociados con la resistencia a la citotoxicidad mediada por células NK. Además, la transferencia de astrocitos transformados dentro de ratones SCID aumento la resistencia a la citotoxicidad mediada por células NK, lo que se sugiere que los cambios específicos en un supresor de tumores (Rb) y un proto-oncogén (Ras) son suficientes para conferir resistencia a la citotoxicidad mediada por células NK en células de glioma y, por tanto, proporcionar una idea de la capacidad de las células tumorales para evadir la respuesta inmune.

## Background

Tumorigenesis is a multiple step process in which genetic alterations drive the progressive transformation of normal cells into highly malignant derivatives with well-known hallmarks [1, 2]. In addition, two “emerging hallmarks” of cancer have recently been proposed, namely deregulation of cellular energetics, and avoiding immune destruction [3]. Furthermore, neoplastic transformation drives genome instability and mutation, and tumor-induced inflammation [3, 4].

The idea that tumors must escape from immune recognition implies that tumors can be destroyed by the immune response [5]. However, some tumors generate an immune suppressive environment, thus evading immune destruction [6]. Gliomas are the most common primary tumors in the brain and are divided into four clinical grades on histopathological and prognosis basis [7]. Several gene expression alterations and chromosomal abnormalities are commonly found in gliomas and, in some instances, these mutations correlate with the clinical grade [8].

In most cancers, the oncogenic Ras is activated, and 20-30 % of all tumors harbor oncogenic point mutations in Ras. Moreover, if Ras is not mutated, such as in gliomas, it is frequently found that the Ras signaling pathway is disrupted [2].

On the other hand, the tumor suppressor Rb regulates cell cycle, inhibiting progression into the S phase, by inactivating the E2F transcription factor, which is critical for DNA replication. The Cancer Genome Atlas (TCGA) project has shown that CDKN2A/p16-CDK4/6-RB pathway is altered in nearly 80 % of primary GBMs with the most frequent genetic alterations being CDKN2A gene deletion or mutation, CDK4 amplification, and RB1 mutation or deletion [9, 10].

Natural killer cells (NK) are regarded as the first line of defense against tumors [11]. Therefore, taking advantage of an oncogenic Ras expression and *Rb* inactivation-based *in vitro* model of gliomagenesis, as previously reported [12], we explored whether these specific genetic alterations induce a cell phenotype compatible with glioma cell evasion from NK cell-mediated cytotoxicity. In addition, *in vitro* transformed glioma cells were injected into SCID mice and after tumor growth, two cell lines that survived the cytotoxic effect of mice NK cells were also analyzed and showed increased resistance to NK cell-mediated cytotoxicity. Together, our results suggest that overexpression of mutated Ras, down-regulation of *Rb*, or both genetic traits, confer *in vitro* resistance to NK cells and that *in vivo* NK cell-based selective pressure, selected cells with an increased *in vitro* resistance to NK cells.

## Results

### Characterization of *in vitro* transformed astrocytes

Four types of transformed astrocytes were obtained, named as *cRb*^*loxP/loxP*^, Ras^V12^, c*Rb*^*−/−*^, and c*Rb*^*−/−*^/Ras^V12^. Overexpression of Ras induced cell morphology heterogeneity, including elongated cytoplasm and multinucleated cells, and loss of contact inhibition, all characteristic traits of transformed cells. Primary astrocytes (c*Rb*^*loxP/loxP*^), and *Rb*-deficient astrocytes showed no significant morphologic alterations. All the four cell types tested positive for glial fibrillary acidic protein (GFAP), thus demonstrating their glial nature.

As expected, the presence of Ras^V12^ was observed in the cell types in which Ras^V12^ was constitutively activated (Ras^V12^, and c*Rb*^*-/ -*^*/*Ras^V12^), and the lack of the Rb protein was observed in the cell types in which *Rb* gene was removed by the Cre recombinase (c*Rb*^*−/−*^, c*Rb*^*−/−*^*/*Ras^V12^). Likewise, the activation of the DNA damage response, as assessed by the expression of p53, p-p53, and p-H2AX was higher in the c*Rb*^*−/−*^*/*Ras^V12^ cells.

**Fig. 1:**
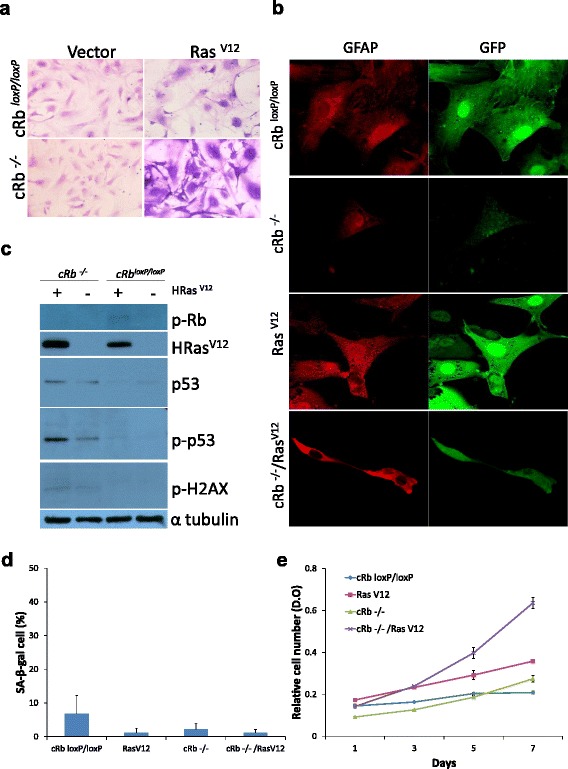
Characterization of *in vitro* transformed astrocytes. (**a**) Morphological changes of astrocytes stained with violet crystal, (**b**) expression of GFAP and GFP in transformed astrocytes, by immunofluorescence, (**c**) expression of pRb, p53, p-p53, RasV12 and p-H2AX, by Western blot with specific antibodies, (**d**) cell senescence, as assessed by the percentage of SA-β-galactosidase positive cells, (**e**) cell proliferation rate, as assessed by violet crystal violet uptake. All images are representative of at least three independent experiments

No significant cell senescence, as assessed by SA-β-gal expression was observed in any of the transformed astrocytes, and the maximal proliferation rate was observed in the c*Rb*^*−/−*^*/*Ras^V12^ cells. All these results, shown in Fig. 1, were very similar to the reported by Seoane et al. [12], in addition to confirming previous data, the cells that were specifically derived for this work were characterized.

### Rb mutation and overexpression of Ras modify the expression of ligands for NK cell receptors

**Fig. 2:**
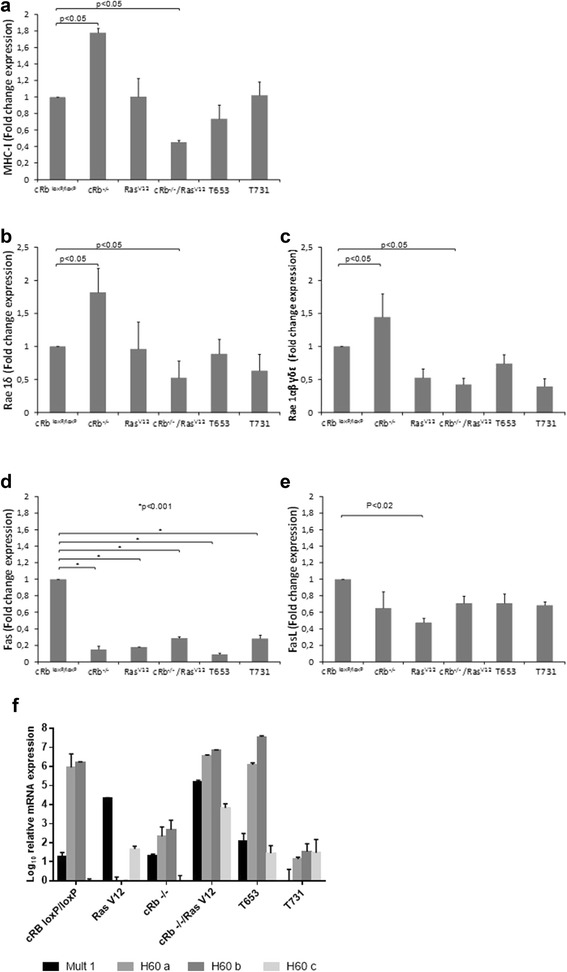
Ras overexpression and *Rb* deletion *in vitro*, induce differential expression of MHC-I, Rae1α, Rae1αβγδε, Fas, FasL, Mult1, H60a, H60b and H60c. Murine astrocytes were transformed *in vitro* for the overexpression of Ras, the deletion of *Rb* or both. In addition, two cell lines were derived from tumors that develop in SCID mice after transplantation of *in vitro* transformed astrocytes (T653, and T731). Expression of cell surface molecules, as indicated, was assessed by flow cytometry after cell staining with specific antibodies, as described in material and methods. Mean fluorescence intensity numerical values were normalized and given a value of 1.0 for the parental cell (c*Rb* ^*loxP/loxP*^), and the fold change of expression for the transformed astrocytes was then calculated. The expression for (**a**) MHC-1, (**b**) Rae1, (**c**) Rae1αβγδε, (**d**) Fas, and (**e**) FasL is shown. Results represent the media +/− S.D. from three independent experiments. mRNA expression for Mult1, H60a, H60b and H60c (**f**) was assessed by Real Time PCR using specific primers and SYBR Green dye as described in material and methods. All expression levels of interested genes were normalized to the housekeeping gene β actin. Gene expression values were then calculated based on the ΔΔCt method. Results represent the media +/− S.D. from three triplicates. Statistical significance was set at p < 0.05

To gain some insight into the mechanisms that confer tumor cells the ability to avoid immune destruction. We tested the expression of defined ligands for NK cell receptors, including MHC class I (an NK inhibiting receptor) and Rae1δ, Rae1αβγδε, mult1, H60a, H60b, H60c, as well as two molecules involved in programed cell death (Fas, and FasL); MHC class I, Rae1δ, and Rae1αβγδε, were analyzed by Western blot, whereas mult1 and H60a, H60b and H60c expression was analyzed by real time PCR. Figure 2a shows the normalized expression of MHC class I (a), Rae1δ (b), Rae1αβγδε (c), Fas (d), and FasL (e). Ligand expression is presented as the fold change, as compared to the expression of untransformed astrocytes. MHC class I expression was higher in c*Rb*^*−/−*^ and lower in *Rb*^*−/−*^*/*Ras^V12^ astrocytes; Rae1δ expression was higher in c*Rb*^*−/−*^, and lower in *Rb*^*−/−*^*/*Ras^V12^ astrocytes; Rae1αβγδε expression was higher in *cRb*^*−/−*^, and lower in *Rb*^*−/−*^/Ras^V12^ astrocytes; FasL expression was lower in Ras^V12^ astrocytes, and Fas expression was lower in all transformed astrocytes. Figure 2b shows the mRNA expression of mult-1, H60a, H60b, and H60c. Mult1 expression was higher in Ras^V12^ and *Rb*^*−/−*^*/*Ras^V12^ than in T731 astrocytes, H60a and H60b expression was higher in *cRb*^*loxP/loxP*^, c*Rb*^*−/−*^, *Rb*^*−/−*^/Ras^V12^ and T731 astrocytes than in Ras^V12^ astrocytes, H60b expression was higher in *cRb*^*loxP/loxP*^, c*Rb*^*−/−*^, *Rb*^*−/−*^/Ras^V12^ and T731 astrocytes than in Ras^V12^ astrocytes, and H60c expression was higher in Ras^V12^, *Rb*^*−/−*^/Ras^V12^, T651 and T731 than in *cRb*^*loxP/loxP*^ and c*Rb*^*−/−*^astrocytes.

### Rb mutation and overexpression of Ras induces resistance to NK cell mediated cytotoxicity

**Fig. 3:**
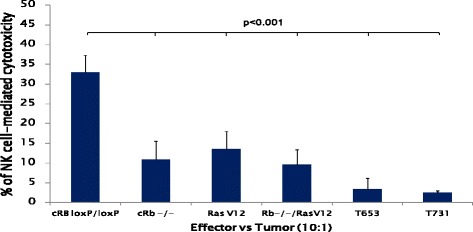
Ras overexpression and *Rb* deletion induce resistance to NK cell-mediated cytotoxicity in *in vitro* transformed astrocytes. NK cells were purified from C57 mice spleens and co-cultured with *in vitro* transformed astrocytes (GFP expressing cells) to an effector target ratio of 10:1. After 4 h of incubation at 37 °C, cells were stained with 7-AAD and the percentage of dead cells in the GFP+ population (target cells) was calculated, and referred to as the % of NK cell-mediated cytotoxicity. Results show the media +/− S.D. of four independent experiments. In all cases the % of NK cell-mediated cytotoxicity was lower in transformed cells than in the parental (c*Rb* ^*loxP/loxP*^) cells (p < 0.001)

Transformed astrocytes were exposed to murine NK cells in order to assess their susceptibility to NK cell-mediated cytotoxicity. Figure 3 shows that in an effector to target ratio of 10:1, approximately 30 % of untransformed astrocytes were lysed by NK cells, whereas all transformed astrocytes tested were more resistant to NK cell-mediated cytotoxicity, as shown by the reduced percentages of specific lysis.

### Ras^V12^ and *cRb*^*−/−*^*/*Ras^V12^ astrocytes produce tumors in FVB immunocompetent mice

**Fig. 4:**
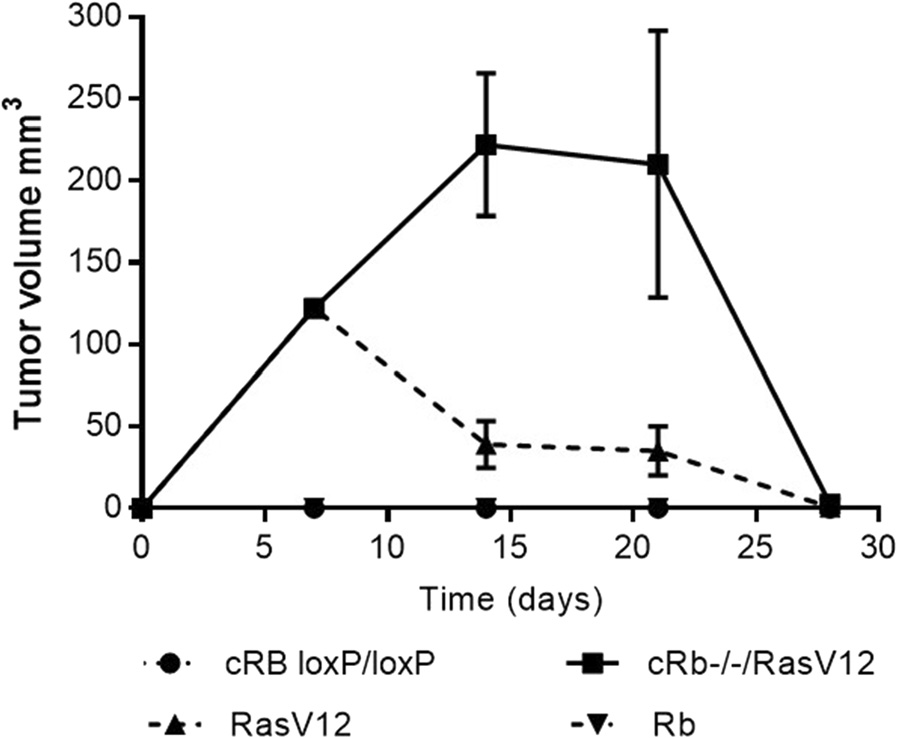
Ras overexpression and *Rb* deletion *in vivo* produce tumours in a syngeneic model. 1x10^6^ cRb^loxP/loxP^, Ras^V12^, cRb^−/−^, or cRb^−/−^/Ras^V12^ transformed astrocytes were subcutaneously injected in FVB immunocompetent mice. Tumours were measured weekly and their volumes (in cubic millimeters) were reported in the graph during 28 days post-implant. Results show the media +/− S.D. of 10 mice

To assess the capacity of transformed glioma cell lines to produce tumors in syngeneic immunocompetent mice, we injected 1x10^6^ cells from either *cRb*^*loxP/loxP*^, Ras^V12^, c*Rb*^*-/* -^, or c*Rb*^*−/−*^/Ras^V12^ astrocytes in FVB mice (10 animals per group). As shown in Fig. 4, only Ras^V12^ and c*Rb*^*−/−*^/Ras^V12^ astrocytes formed tumors. Tumors formed by injection with Ras^V12^ cells grew during the first week, and after that, all the tumors formed were reabsorbed. c*Rb*^*−/−*^/Ras^V12^ tumors grew during the first two weeks and then began to involute until the fourth week. At about this time, tumors were completely reabsorbed.

### Effect of Rb deleted and/or RAS^V12^ overexpressed tumor cells on immune cell phenotype in the peripheral blood

**Fig. 5:**
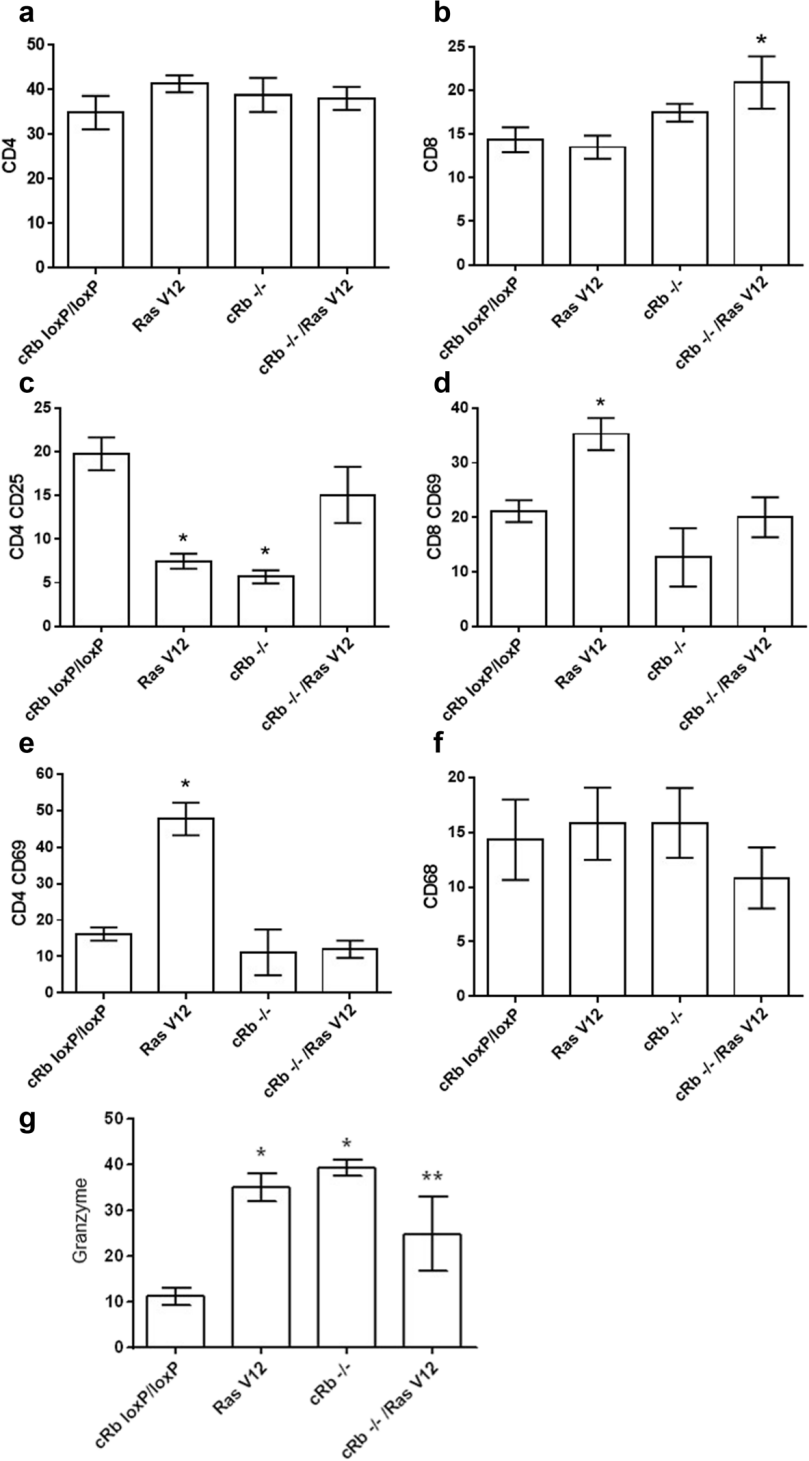
Flow cytometry analysis of peripheral blood. (**a**) % of T helper lymphocytes (CD4+) from mice implanted with transformed astrocytes, (**b**) % of T cytotoxic lymphocytes (CD8+) from mice implanted with transformed astrocytes, (**c**) % of late activate T helper lymphocytes (CD4+/CD25+) from mice implanted with transformed astrocytes, (**d**) % of early activated T cytotoxic lymphocytes (CD8+/CD69+) from mice implanted with transformed astrocytes, (**e**) % of early activated T helper lymphocytes (CD4+) from mice implanted with transformed astrocytes, (**f**) % of macrophages (CD68+) from mice implanted with transformed astrocytes, (**g**) % of cells containing granzyme from mice implanted with transformed astrocytes. Results show the media +/− S.D. of 10 mice

In order to analyze the immune response against transformed glioma cell lines with Rb deletion and/or RAS^V12^ overexpression in an homologous syngeneic model of tumor transplantation, the percentages of different immune cell subpopulations were quantified in the peripheral blood of mice in which tumor cells had been injected 28 days earlier. Figure 5 shows that mice injected with the c*Rb*^*−/−*^/Ras^V12^ astrocytes increase the percentage of cytotoxic CD8^+^ lymphocytes, as compared to the other groups of mice (*p* = 0.026); a significant reduction in the percentage of regulatory CD4 + CD25+ lymphocytes was observed in mice injected with Ras^V12^ or c*Rb*^*−/−*^ (*p* = 0.001). In addition, the group implanted with Ras^V12^ cells developed a significant increment in the percentage of activated T cells, both CD4 + CD69+ and CD8 + CD69+ cells (*p* = 0.016 and *p* = 0.001 respectively). In all groups of transformed glioma cell line-injected mice, the percentage of granzyme-expressing cells increased as compared to the mice injected with *cRb*^*loxP/loxP*^ astrocytes.

## Discussion

The immune system is thought to be constantly surveying for the arising of malignant cells that would in turn be eliminated by the immune response [13]. An “emerging hallmark” of cancer states that tumor cells are capable of avoiding immune destruction [3].

In gliomas from human origin, the formed tumor is immunosuppressive [14, 15]. How these tumors reach that immunosuppressive characteristic at the early stages of malignant transformation, and whether specific mutations are associated with the ability to escape from the immune response is poorly understood. Here, by using a previously reported *in vitro* model of gliomagenesis [12], we tested the hypothesis that defined changes in the expression of a proto-oncogene (Ras) and a tumor suppressor (*Rb*) confer tumor cells the ability to avoid immune destruction. In particular, we addressed the resistance to NK cell-mediated cytotoxicity. Since NK-cell mediated cytotoxicity is dependent on the tumor cell membrane expression of several ligands that upon engagement with specific NK cell receptors either inhibit or activate NK cell cytotoxic function, the expression of MHC class I (ligand for inhibiting NK receptor Ly49D), Rae1δ, Rae1αβδγε, mult1, H60a, H60b, and H60c (ligands for NKG2D, an activating NK cell receptor) [16–19], as well as of Fas and FasL (molecules involved in programed cell death) was assessed *in vitro* transformed astrocytes. In gliomas from human origin, deregulated expression of MHC-I has been associated with NK cell-mediated cytotoxicity [20], and stem cells from glioma patients do not express protective levels of MHC-I molecules, but they express several ligands that activate NK cells [21].

These studies assessed the susceptibility of both stem cells and tumor cells to NK cell-mediated cytotoxicity, after the onset of malignant transformation and therefore after immunoediting had likely already taken place [5, 22]. Although, down-regulation of MHC-I makes tumor cells susceptible to NK cell-mediated cytotoxicity [22], high expression of MHC-I does not necessarily guarantee resistance to NK cells [23]. Here, we assessed the expression of MHC-I on various *in vitro* transformed astrocytes that therefore were not subjected to any immune-based selective pressure. Results showed a significant increase in the expression of MHC-I in the cRb^−/−^ cells and a significant decrease in the cRb^−/−^/Ras^V12^ cells (Fig. 1a). When Rb^−/−^/Ras^V12^ cells were inoculated into SCID mice, and the T731 tumor cell line was derived, the expression of MHC-I was similar to that of cRb^loxP/loxP^ cells (Fig. 1a). This MHC-I expression recovery may be the result of selective pressure exerted by NK cells in the SCID mice that selected cells with the highest levels of MHC-I, conferring them an advantage to evade the NK cell anti-tumor response. In this regard, the T731 cell line also showed the lowest percentage of NK cell-mediated cytotoxicity (Fig. 2). However, the lower expression of MHC-1 observed in *Rb*^*−/−*^*/*Ras^V12^ cells, and the higher expression of Rae1α and Rae 1αβγδε observed in c*Rb*^*−/−*^ cells seem to be in contradiction with their increased resistance to NK cells. Rae1 expression is low or absent in normal tissues and it is constitutively expressed on some tumor cells. Upon engagement with the NKG2D receptor on NK cells, it activates their cytotoxic activity [24, 25]. Rae1 expression has been associated with cell proliferation [26], and Rae1 gene family members are activated by the E2F transcription factor, which plays a central role in regulating cell cycle entry [27]. A significant increase in the expression of Rae1δ and Rae1αβγδε was found in *Rb*^*−/−*^ astrocytes, as compared with that of parental c*Rb*^*loxP/loxP*^ astrocytes (Fig. 2b and c). Ras induces the expression of Rae1 [28]. However, in this case, overexpression of Ras (Ras^V12^ astrocytes) did not correlate with an increase in Rae1δ or Rae1 αβγδε expression. Moreover, in c*Rb*^*−/−*^*/*Ras^V12^ astrocytes a significant reduction in the expression of both Rae1δ, and Rae1αβγδε was observed (Fig. 1). The T653 and T731 astrocyte lines did not show significant changes in Rae1δ, or Rae1αβγδε expression, as compared with parental c*Rb*^*loxP/loxP*^ astrocytes. This would suggest that loss of Rb promotes the expression of Rae1δ, and Rae1αβγδε. However, since these two ligands would engage with the NK cell activating NKG2D receptor, increased NK cell-mediated cytotoxicity against cRb^−/−^ astrocytes, as compared to that of c*Rb*^*loxP/loxP*^ astrocytes would be expected. This was clearly not the case, since the percentage of NK cell-mediated cytotoxicity was in fact lower than that for c*Rb*^*loxP/loxP*^ astrocytes (Fig. 3). The mRNA expression of four other ligands for NKG2D cell receptors was also assessed (mult1, H60a, H60b, and H60c). These ligands are poorly expressed on most normal cells, but are upregulated on tumor cells, which is keeping with the finding that mult1 mRNA expression was comparatively higher in RasV^12^ and cRb^−/−^/ RasV^12^ cells than in cRb^loxP/loxP^ cells. On the other hand, the mRNA expression of mult1 was similar between cRb^loxP/loxP^ and cRb^−/−^ cells, thus suggesting that transformation due to RasV^12^ over expression but not to Rb deletion would render transformed astrocytes more susceptible to NK cytotoxicity. Again, this was not the case, since both RasV^12^ and cRb^−/−^/ RasV^12^ astrocytes were more resistant to NK cytotoxicity. However, in analyzing the mRNA expression of H60a and H60b, results evident that their expression is lower in RasV^12^ and cRb^−/−^, as compared to cRb^loxP/loxP^ astrocytes. In addition, mRNA expression of H60a and H60b is similar in cRb^−/−^/ RasV^12^ and cRb^loxP/loxP^ astrocytes. Therefore H60a and H60b expression would imply resistance to NK cell cytotoxicity, as it actually happens.

The molecule Fas induces cell death upon engagement with FasL (Fas ligand) [29]. FasL is expressed on activated T and NK cells [30] and thus FasL induces apoptotic cell death on Fas-expressing cells [28]. Accordingly, a decrease in Fas expression would protect cells from cell-mediated cytotoxicity. The loss of Fas expression has been observed in melanoma, breast cancer, leukemia, and lymphoma cells [31–33]. A variety of malignant tumors show increased expression of FasL, thus allowing tumor cells to induce apoptosis on cytotoxic cells, in a process known as “tumor counterattack” [34–36]. Here we showed that overexpression of Ras, deletion of *Rb*, or both, are sufficient to decrease the expression of Fas (Fig. 1d). Lower expression of Fas was concomitant to increased resistance to NK cell-mediated cytotoxicity (Fig. 2). No significant increase in the expression of FasL was observed in any of the transformed astrocytes. The FasL expression data suggest that genetic alterations other than just Ras overexpression and *Rb* deletion are required for the “tumor counterattack” phenotype acquisition. It is tempting to speculate that this phenotype only takes place after acquisition of resistance to NK cells and further genome instability, a property of neoplastic transformation [3].

Additional experiments were designed to evaluate the tumor growth in a syngeneic model (FVB mice) and the possible contribution of other immune cells in the tumor implantation outcome. cRb^−/−^/Ras^V12^, followed by cRb^−/−^ astrocytes were successfully implanted. This could be explained in part by the mRNA expression of H60a and H60b, as mentioned before, and the consequent resistance to NK cell-mediated cytotoxicity (Fig. 3). However, the expression of other ligands for NKG2D is contradictory, mult1 for instance. If tumor implantation can be attributed to resistance to NK cell cytotoxicity, tumor remission could then be attributed to the increase in the percentages of CD8^+^ and granzyme^+^ cells in the case of cRb^−/−^/Ras^V12^ tumor, and to the increase in the percentages of CD8^+^CD69^+^, CD4^+^CD69^+^ and granzyme^+^ cells, and to the decrease in the percentage of regulatory CD4^+^CD25^+^ cells in the case of Ras^V12^ tumor. It is worth noting that the most resistant tumor is the one with the two mutations and also the one that after 28 days post implantation only induced an increase in the percentage of CD8^+^ and granzyme^+^ cells as compared with the Ras^V12^ that induces phenotype changes more consistent with anti-tumor immunity.

Taken together, our results suggest that Ras overexpression and *Rb* deletion are sufficient for the malignant transformation of astrocytes and that these genetic alterations confer transformed cells resistance to NK cell-mediated cytotoxicity, by altering the expression of NK cell receptor ligands, such as the higher expression of MHC-I observed in c*Rb*^*−/−*^ cells, or the lower expression of Rae1α and Rae1αβγδε observed in *Rb*^*−/−*^*/*Ras^V12^ cells, and also by altering the expression of cell death associated molecules such as Fas, as observed in Ras^V12^ cells. Its seems that the innate immune system deals with small antigenic differences between the normal and transformed tumor cells that allow tumor cells to initiate proliferation and to develop a number of oncogenic stages, however further analyses will be required.

## Methods

### Cell culture and cell proliferation assays

Astrocytes were isolated from 3 days old *Rb* floxed mice, as previously described [12]. Animal care and use of all experimental animals were performed in accordance with institutional ethical guidelines. In order to introduce an active *Ras* allele or to promote the *Rb* loss in astrocytes, *Phoenix-*Eco packaging cells (a kind gift from G.P. Nolan) were transfected with empty pBABE, pBABE-HRas^V12^, empty PIG, and PIG-CRE retroviral plasmids (a kind gift from P.P. Pandolfi), and *in vitro* transformation was achieved by retroviral infection. The resulting cells were denominated; c*Rb*^*loxP/loxP*^, Ras^V12^, *cRb*^*−/−*^*,* and *cRb*^*−/−*^*/*Ras^V12^. The T653 and T731 cell lines were derived from tumors formed by inoculation of Ras^v12^ and *cRb*^*−/−*^*/*Ras^V12^ cells in SCID mice, as previously shown [12]. A feature of the SCID mice is a deficiency in the recombination of genes needed for full maturation of T and lymphocytes. However, SCID mice harbor functional NK cells. Cells were maintained in Dulbecco Modified Eagle Medium (DMEM) (Sigma-Aldrich, St Louis, MO) supplemented with 10 % FBS and antibiotic-antimycotic solution (Bio West, Nuaillé, France). For cell growth analysis, 5x10^3^ cells were plated into 24-well culture plates (Corning, NY, USA) and then fixed with methanol/acetic acid (3:1) on days 1, 3, 5, 7 of culture, for subsequent staining with crystal violet (0.1 % in PBS) and distaining with 10 % acetic acid. The relative cell number was assessed by spectrophotometry.

### Syngeneic model

The performance of tumor growth in the syngeneic model was evaluated by subcutaneously injecting 1x10^6^ cRb^loxP/loxP^, Ras^V12^, cRb^−/−^, or cRb^−/−^/Ras^V12^ astrocytes previously obtained from FVB mice (*n* = 10). Tumours from all animals (10 per group) were measured weekly, and their volumes (in cubic millimeters) were determined with the formula 6/π × L × W × H. After 28 days, animals from all groups were anaesthetized and sacrificed by exsanguination.

### Flow Cytometry of T lymphocytes, macrophages and granzyme

Immunofluorescence using monoclonal antibodies was used to determine the percentages of CD68+, CD4+, CD8+, CD4+/CD25+, CD8/CD25+, CD8+/CD69+, CD4+/CD69+ and granzyme^+^ cells in the peripheral blood samples, (Biolegend, USA). Briefly, 30 μl of blood were incubated for 30 min with 5 μl of the corresponding monoclonal antibody (1:100 dilution) afterwards, 200 μl of lysis solution were added (Becton Dickinson, California), incubated in darkness for 10 min and washed twice with 0.1 M PBS (pH 7.2), 0.1 % BSA and 0.1 % NaN3. The cells were then fixed in 1 % paraformaldehyde solution and stored at 4 °C until examination by flow cytometry (FACSCalibur, Becton Dickinson) using the Cell Quest software. 10,000 events in the region corresponding to lymphocytes were analyzed. From this region, the percentage of positive cells from each sample was determined. Results were expressed as means (±SD) for each experimental group.

### Senescence assay

Cell senescence was assessed by the expression of β-galactosidase, by using a β-galactosidase staining kit (Cell Signaling, Danvers, MA). Cells (5x10^3^) were plated in triplicate into 24-well culture plates (Corning, NY, USA) and then fixed on day 6 of culture for subsequent β-galactosidase staining.

### Immunoblot

Cell proteins were extracted in RIPA buffer (1 % Nonidet P-40, 0.5 % sodium deoxycholate, 0.1 % SDS in PBS) in the presence of 40 μg/ml of aprotinin, 10 μg/ml PMSF and 100 mM orthovanadate. 40 μg of total protein were separated by 8 % or 12 % SDS-PAGE and transferred to nitrocellulose membranes. Western blot were developed with antibodies against p-p53 (Cell signaling, Danvers, MA), p-H2AX (Millipore, MA, USA), p53 (cell signaling, Danvers, MA), pan-Ras-^V12^ (Calbiochem, MA, USA), p-Rb (BD Biosciences, San Jose, CA, USA), or α-tubulin (Sigma-Aldrich, St Louis, MO).

### Phenotypic analysis (by Immunofluorescence)

Cells were growth in 8-well polystyrene chambers (BD Falcon, San Jose, CA, USA) until 80 % confluence, fixed with 4 % paraformaldehyde in PBS, blocked with 1 % BSA in PBS, and labeled overnight at 4 °C with anti-GFAP antibody (Millipore, MA, USA) followed by Alexa fluor-594-conjugated anti-rabbit IgG (Invitrogen). Cells were mounted in Vectashield (Vector, CA, USA) and analyzed by confocal microscopy (LSMS Pascal, Zeiss).

### Phenotypic analysis (by Flow cytometry)

Cells were suspended in blocking buffer (0.5 % BSA/ 2 mM EDTA, in PBS) and then labeled with fluorochrome-conjugated antibodies: PE-conjugated anti-Fas (BD Biosciences, San Jose, CA, USA), PE-conjugated anti-FasL (Biolegend, San Diego, CA) (1 μg/ml), or anti-MHC-I (Biolegend, San Diego, CA), anti-RAE-1δ (Biolegend, San Diego, CA), anti-Rae-1αβγδε (scbt, CA, USA) (1:50), followed by APC-conjugated anti-mouse IgG (scbt, CA, USA) or APC-conjugated anti-rabbit IgG (scbt, CA, USA) (1:50) secondary antibodies, as appropriate. Cell membrane expression of these molecules was assessed by Flow Cytometry (FACS Aria III, BD Biosciences). Raw data was further analyzed by using Flow Jo software (Tree Star, Inc. Ashland OR).

### NK cytotoxicity assays

NK cells were purified from mice spleens (C57 strain), hosted in the INNN animal house, in accordance with institutional guidelines, by using the NK cell isolation kit II (Myltenyi Biotech, Germany), following the manufacturer”s instructions. NK cell cytotoxicity against tumor cells was evaluated by using Lecoeur et al. method [37]. Since the tumor cells here used express green fluorescent protein (GFP) due to the transformation procedure, there was no need to label them. Isolated NK cells and tumor cells were co-cultured in 10 % FBS/DMEM at 1:10 target/effector cell ratio, for 4 h. After which, cells were labeled for 15 min with 7-aminoactinomycin D (BD Pharmingen, San Jose, CA, USA) at a final concentration of 20 μl/ml. Flow cytometry analysis was used to calculate the percentage of green fluorescent cells (tumor cells) that were stained by 7-AAD (dead cells). Results are expressed as the percentage of specific lysis calculated by the following formula:

$$ \%\ \mathrm{specific}\ \mathrm{lysis} = 100 \times \left(\%\kern0.5em \mathrm{sample}\ \mathrm{lysis} - \%\ \mathrm{basal}\ \mathrm{lysis}\right)/100 - \%\ \mathrm{basal}\ \mathrm{lysis} $$

### Quantitative polymerase chain reaction

Total RNA was extracted via the phenol/chloroform method using TRIzol reagent (Invitrogen). Quantitative polymerase chain reaction (PCR) was performed using EXPRESS One-Step SYBR® GreenER™ Kit (Invitrogen, USA). Emissions from the SYBR Green reporter dye were monitored with an ABI Prism 7500 Real Time PCR (Applied Biosystems). The primer sequences used were as follows:

Mult1, 5′-CAATGTCTCTGTCCTCGGAA-3′ (sense), Mult1, 5′-CTGAACACGTCTCAGGCACT-3′ (antisense); H60a, 5′-TGCCTGATTCTGAGCCTTTTCA-3′ (sense), H60a, 5′-ATTCACTGAGCACTGTCCATGTAGAT-3′ (antisense); H60b, 5′-AGCCTTTTGGTCCTGCTGAAT-3′ (sense), H60b, 5′-ATGTTTTTTATCACCAAAATCAAGGAGT-3′ (antisense); H60c, 5′-CTTCTCTTGATCCTGGAGTCCTGTAGT-3′ (sense), H60c, 5′-GAGAGTCTTTCCATTCACTGAGCAC-3′ (antisense); β-actin, 5′-TTCTACAATGAGCTGCGT-3′ (sense), β-actin, 5′-ATCACAATGCCTGTGGTA-3′ (antisense). All expression levels of interested genes were normalized to the housekeeping gene β-actin. Gene expression values were then calculated based on the ΔΔCt method.

### Statistical analyses

Data was summarized as arithmetic means and standard deviations (SD). One-way analysis of variance (ANOVA) and post-hoc (Tukey) test were conducted. Statistical significance was set at p < 0.05 in a two-sided test. SPSS software package V 18.0 for Windows; (SPSS Inc., Chicago, IL) was employed for data analysis.

## Abbreviations

Rb: Retinoblastoma
NK: Natural killer
MHC: Major Histocompatibility Complex
